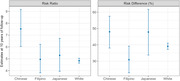# Effect of incident stroke on the risk of dementia over 10 years of follow‐up in a cohort of Asian American and White older adults in Northern California

**DOI:** 10.1002/alz.092080

**Published:** 2025-01-09

**Authors:** L. Paloma Rojas‐Saunero, Yixuan Zhou, Eleanor Hayes‐Larson, Yingyan Wu, Roch A Nianogo, Rachel A. Whitmer, Paola Gilsanz, Holly C Elser, Elizabeth Rose Mayeda

**Affiliations:** ^1^ UCLA Fielding School of Public Health, University of California, Los Angeles, CA USA; ^2^ University of California, Davis School of Medicine, Sacramento, CA USA; ^3^ Kaiser Permanente Northern California Division of Research, Oakland, CA USA; ^4^ Hospital of the University of Pennsylvania, Philadelphia, PA USA

## Abstract

**Background:**

Acute stroke may increase dementia risk. Previous work has not accounted for time‐varying covariates that could increase risk of stroke and dementia over time, and there has been very limited evidence on the effect in Asian Americans. We aimed to estimate the effect of incident stroke on dementia risk over 10 years of follow‐up among Asian American and White older adults in Northern California considering time‐varying covariates.

**Methods:**

We included Chinese (n = 6034), Filipino (n = 4649), Japanese (n = 3099), and White (n = 133208) members of Kaiser Permanente who participated in the Research Program on Genes, Environment, and Health and the California Men’s Health Study surveys (2002‐2009), with linked electronic health records (EHR) data until 2020. Inclusion criteria included age 60‐89 years old and no history of stroke or dementia at the time of survey (baseline). Incident stroke (ischemic and hemorrhagic) and incident dementia diagnosis were defined based on ICD codes from EHR. To ensure comparability between those with and without incident stroke at all time points before incident stroke, we fit annual inverse probability treatment weights (IPTW) to adjust for confounding, including baseline sociodemographic covariates and time‐varying (annual) cardiovascular covariates. We also fit censoring weights (IPCW) so that those who remained alive over follow‐up are representative of those who died. We estimated the risk ratio (RR) and risk difference (RD) for incident stroke and subsequent dementia from weighted Kaplan‐Meier survival estimators at 10 years of follow‐up for each racial/ethnic group. Sensitivity analyses examined ischemic stroke as the outcome.

**Results:**

The cause‐specific cumulative incidence of stroke at 10 years of follow‐up ranged between 10.9% (95%CI: 10.0%, 11.8%) for Chinese and 14.1% (95%CI: 12.7%, 15.5%) for Japanese participants. After adjusting for confounding and censoring, the RR for the effect of incident stroke on subsequent dementia was 5.6 (95%CI: 4.2, 7.3) for Chinese, 3.8 (95%CI: 2.7, 5.1) for Filipino, 4.9 (95%CI: 3.0‐6.6) for Japanese participants, and 3.3 (95%CI: 3.1‐3.5) for White participants. Confidence intervals between groups mostly overlapped (Figure). Restricting analysis to ischemic stroke only yielded similar results.

**Conclusion:**

The effect of stroke on risk of dementia is large among Asian American and White older adults.